# Functions of N6-methyladenosine in cancer metabolism: from mechanism to targeted therapy

**DOI:** 10.1186/s40364-023-00483-8

**Published:** 2023-04-13

**Authors:** Jiayi He, Furong Liu, Zhanguo Zhang

**Affiliations:** 1 Hepatic Surgery Center, Tongji Hospital, Tongji Medical College, Huazhong University of Science and Technology, Wuhan, Hubei 430030 China; 2 Hubei Key Laboratory of Hepato-Pancreato-Biliary Diseases, Wuhan, Hubei 430030 China

**Keywords:** N6-methyladenosine, Cancer, Metabolic reprogramming

## Abstract

N6-methyladenosine (m6A) is the most abundant modification of eukaryotic mRNA and is involved in almost every stage of RNA metabolism. The m6A modification on RNA has been demonstrated to be a regulator of the occurrence and development of a substantial number of diseases, especially cancers. Increasing evidence has shown that metabolic reprogramming is a hallmark of cancer and is crucial for maintaining the homeostasis of malignant tumors. Cancer cells rely on altered metabolic pathways to support their growth, proliferation, invasion and metastasis in an extreme microenvironment. m6A regulates metabolic pathways mainly by either directly acting on metabolic enzymes and transporters or indirectly influencing metabolism-related molecules. This review discusses the functions of the m6A modification on RNAs, its role in cancer cell metabolic pathways, the possible underlying mechanisms of its effects and the implication of this modification in cancer therapy.

## Introduction

N6-methyladenosine (m6A), an epigenetic modification, has been intensely studied in recent years. m6A is the predominant chemical modification on eukaryotic mRNA [1] and is involved in almost every stage of the RNA life cycle, including RNA splicing, nuclear output, decay, folding and translation [2, 3]. Significantly, m6A RNA modification plays a critical role in a variety of physiological processes and human diseases, such as tissue development [4], cell differentiation and pluripotency [5, 6], DNA damage repair [7], obesity [8], infertility [9] and even cancer progression [10]. Many studies have confirmed that m6A regulates the function of various RNA classes, including messenger RNAs (mRNAs) [11], ribosomal RNAs (rRNAs) [12], transfer RNAs (tRNAs) [13], long noncoding RNAs (lncRNAs) [14], microRNAs (miRNAs) [15–17], and circular RNAs (circRNAs) [18]. Due to the diversity of RNA modification sites, m6A can either facilitate or suppress the progression of many diseases, including human cancers. Recently, the most widely employed m6A detection technique has been methylated RNA immunoprecipitation sequencing [19], which can be used to identify m6A methylation hypermethylated regions. In contrast to methylated RNA immunoprecipitation sequencing (MeRIP-Seq/m6A-Seq), liquid chromatography–mass spectrometry (LC‒MS) is used to measure the overall m6A level of mRNA. An increasing number of novel methods, such as m6A individual-nucleotide resolution crosslinking and immunoprecipitation (miCLIP) [20], site-specific cleavage and radioactive labeling followed by ligation-assisted extraction and thin-layer chromatography (SCARLET) [21], have been developed to identify the diverse functions affected by the m6A modification.

Metabolic reprogramming, associated with a variety of deregulated metabolic pathways [22] and metabolic enzymes, is a main feature of cancer [23]. The metabolic pathways in cancer cells with increased proliferation are better adapted to survival in the extremely hypoxic and nutrient-altered microenvironment. In addition to aerobic glycolysis, best known as the Warburg effect [24], altered fatty acid metabolism, aberrant amino acid metabolism and mitochondrial metabolism have recently gained increasing attention in the field of cancer research.

Our review describes recent studies on the various functions of the m6A modification in cancer metabolic pathways, together with the pivotal biological roles of m6A in eukaryote cells. It then presents a discussion on the underlying mechanisms in cancer metabolism. These findings provide fresh insights into early cancer diagnosis and clinical therapies.

## The m6A

Studies have revealed that approximately 0.1–0.4% of adenylate residues in mammals are modified with the m6A, accounting for approximately one-half of all methylated ribonucleosides [1, 25]. m6A, mostly deposited on the common DRm6ACH (D = A/G/U, R = G/A, H = A/C/U) motif [26–29], is enriched at the beginning of 3’-untranslated regions (3’-UTRs), proximal to translation termination codons and within internal long exons [19, 30]. The m6A modification is deposited, removed and identified by several writers, erasers and readers, respectively. The methyltransferase complex, termed the “writer”, catalyzes the addition of the m6A mark to target RNAs [31]. In contrast, demethylases, namely, “erasers”, remove m6A RNA methyl groups. To perform various downstream biological functions, “readers”, a group of specific proteins, bind to m6A methylation sites. Readers are proteins that specifically recognize and bind to m6A-modified sites, performing different downstream biological functions. Altogether, these enzymes modulate an integrated m6A network and are involved in cancer progression.

## Methyltransferases mediating the m6A modification

m6A is installed by a multicomponent methyltransferase complex that mainly comprises the catalytic subunit METTL3 and many other components, such as METTL14 [32], METTL16 [33, 34], WTAP [35], VIRMA (KIAA1429) [36, 37], RBM15/15B [14] and ZC3H13 [38, 39]. In the 1990s, methyltransferase-like 3 (METTL3), a 70 kD protein, was discovered to be an important element of the m6A methyltransferase complex in human HeLa cells, and it primarily exhibited catalytic activity in eukaryotes ranging from yeast to humans [40, 41]. Homologous to METTL3, methyltransferase-like 14 (METTL14) was found to be located in nuclear plaques, and the two writers together formed a heterodimer. METTL14 is catalytically inactive but plays a significant role in facilitating METTL3 recognition of target RNAs [32, 42]. Methyltransferase-like 16 (METTL16), another m6A methyltransferase, has been reported to alter S-adenosylmethionine (SAM) levels and target precursor mRNAs and noncoding RNAs, including U6 small nuclear RNA [33, 34]. In addition, Wilms’ tumor 1-associated protein (WTAP) is a significant regulatory element in the m6A methyltransferase complex. Structurally, WTAP harbors no catalytic subunit and thus can neither catalyze the m6A modification nor positively affect the METTL3–METTL14 complex in vitro [32]. It has been demonstrated that WTAP is a regulator of METTL3-METTL14 complex accumulation in vivo. In other words, WTAP recruits METTL3 as well as METTL14 to constitute a stable dimer to ensure precise complex localization to nuclear spots [35].

Vir-like m6A methyltransferase associated (VIRMA), also KIAA1429, has been verified to facilitate mRNA methylation in mammalian cells [36]. Specifically, VIRMA preferentially recruits the METTL3/METTL14/WTAP component to 3’-UTRs and near a termination codon and is related to the cleavage factors CPSF5 and CPSF6. Moreover, RNA-binding motif protein 15 (RBM15) and its paralog RBM15B participate in m6A installation by guiding the m6A complex to specific RNA sites [14]. Zinc finger CCCH domain-containing protein 13 (ZC3H13) is important for m6A modification and required for nuclear localization of m6A by linking WTAP with Nito, an mRNA-binding factor [39].

## Demethylases removing the m6A modification

Fat mass and obesity-associated protein (FTO), AlkB Homolog 5 (ALKBH5) and AlkB Homolog 3 (ALKBH3) are erasers that can dynamically reverse m6A RNA modification deposition. Among these three demethylases, FTO and ALKBH5 play major roles in removing the methyl group from the m6A mark. The first discovered demethylase of m6A modification was FTO. Previous studies have shown that FTO is associated with obesity and regulates energy homeostasis [43–45]. As a member of the dioxygenase AlkB family of proteins, FTO depends on Fe(ii) and α-ketoglutarate to catalyze the m6A modification [46]. Similar to FTO, iron(II)/α-ketoglutarate-dependent dioxygenase homolog 5 (ALKBH5) was later confirmed to be the second discovered m6A RNA eraser. Decreased levels of ALKBH5 augment m6A abundance on nuclear RNA, suggesting the reversibility of the m6A modification [47]. In 2017, another study confirmed that mammalian AlkB homolog 3 (ALKBH3) effectively promoted RNA demethylation and that the knockdown of ALKBH3 contributed to the accumulation of methylated RNAs. This research also identified tRNA as a new ALKBH3 substrate [48].

## Proteins binding to RNA m6A sites

The m6A modification of RNA is regulated by the mutual effect between m6A methyltransferases and demethylases. However, subsequent biological processes require the identification of various proteins that bind to specific modification sites. These proteins are “readers,” which recognize m6A modifications alone or in combination. The most noted m6A readers are in the YT521-B homolog.

(YTH) domain family and insulin-like growth factor 2 mRNA-binding protein (IGF2BP) family [49]. The former set includes YTH domain family protein 1/2/3 (YTHDF1/2/3) and YTH domain-containing 1/2 (YTHDC1/2), while the latter family is composed of IGF2BP1/2/3. The m6A binding sites of YTHDF1 cluster around the stop codons, and then YTHDF1 recognizes m6A, interacts with the translation initiation complex and accelerates the translation process of mRNA in mammalian species [50]. In contrast, YTHDF2 promotes mRNA decay. The C-terminus of YTHDF2 selectively identifies m6A-modified mRNA, while the N-terminus recruits the carbon catabolite repression 4-negative on TATA-less (CCR4-NOT) complex and forms bridges between the mRNA and the processing body, thus mediating the decay of select transcripts [51]. YTHDF3 plays a synergistic role in RNA metabolism by interacting with other YTH domain family members. It reinforces the translation of targeted RNAs by cooperating with YTHDF1 [52] and facilitates the degradation of m6A-modified mRNA in the presence of YTHDF2 [53]. Another m6A reader, YTHDC1, was initially shown to facilitate exon inclusion and regulate mRNA splicing by recruiting serine- and arginine-rich splicing factor 3 (SRSF3) or restricting SRSF10 recruitment, bridging nuclear m6A-modified RNA to the nuclear export adaptor protein SRSF3, nuclear RNA export factor 1 (NXF1) and the three-component prime repair exonuclease (TREX) complex, mediating nuclear efflux [54–56]. Additionally, YTHDC1 preferentially identifies m6A residues on X-inactive specific transcript (XIST), which is a long noncoding RNA, indirectly repressing transcriptional [14]. YTHDC2 has been reported to function together with meiosis-specific protein (MEIOC) after binding m6A inside the consensus GGACU motif, reducing mRNA abundance while promoting the translation efficiency of target mRNAs [57]. An increasing number of studies have shown that insulin-like growth factor 2 mRNA-binding protein 1/2/3 (IGF2BP1/2/3) are required for the m6A reading process. The K homology (KH) domains are critical to the capability of these readers to recognize the m6A modification. In contrast to YTHDF2, IGF2BPs are promoters of the stability, storage and translation of mRNAs, highlighting their significant biological roles in gene regulation [49, 58, 59].

Several members of the heterogeneous nuclear ribonucleoprotein (HNRNP) family are also m6A readers, and they are critical to pre-mRNA processing [60]. Binding to m6A sites in certain RNA transcripts, heterogeneous nuclear ribonucleoprotein A2B1 (HNRNPA2B1) induces alternative splicing effects. The interaction between HNRNPA2B1 and the primary microRNA microprocessor complex protein DiGeorge syndrome critical region 8 (DGCR8) enhances the processing of primary miRNAs [61]. This action is called “m6A switching’’. Heterogeneous nuclear ribonucleoprotein C (HNRNPC) and heterogeneous nuclear ribonucleoprotein G (HNRNPG) binding can exert obvious effects on the splicing of m6A-modified mRNAs, whereas m6A can remodel RNA structure to facilitate the binding of HNRNPC and HNRNPG to mediate mRNA abundance and splicing. “m6A-switching’’ is a possible underlying mechanism of m6A wide-ranging physiological functions [2, 62]. Eukaryotic initiation factor 3 (eIF3) is an essential component for initiating cap-independent translation under basal cellular conditions, and it is recruited by m6A in 5’-UTRs and subsequently recruits the 43S complex to promote mRNA translation [63]. During mRNA circularization, eIF3H physically and functionally interacts with the m6A writer METTL3 to facilitate cap-dependent translation [64]. (Table 1)

**Table 1:** Functions of m6A enzymes

Type	Factor	Function	Reference
m6A writer	METTL3	catalyzes m6A methylation	[40, 41]
	METTL14	assists METTL3 in recognizing target RNAs	[32, 42]
	METTL16	catalyzes m6A methylation	[33, 34]
	WTAP	regulates the accumulation of METTL3 and METTL14 to form a stable heterodimer	[35]
	VIRMA (KIAA1429)	recruits METTL3/METTL14/WTAP component to make contact with CPSF5 and CPSF6	[36]
	RBM15/15B	guide m6A complex to specific RNA sites	[14]
	ZC3H13	links WTAP with the mRNA-binding factor Nito	[39]
m6A eraser	FTO	catalyzes m6A demethylation	[46]
	ALKBH5	catalyzes m6A demethylation	[47]
	ALKBH3	promotes RNA demethylation	[48]
m6A writer	YTHDF1	recognizes m6A and interacts with translation machinery to accelerate mRNA translation	[50]
	YTHDF2	selectively identifies m6A-containing mRNA and promote the degradation of m6A-containing transcripts	[51]
	YTHDF3	reinforces the translation of targeted RNAs via cooperating with YTHDF1 and facilitates the degradation of m6A-modified mRNA through association with YTHDF2	[52, 53]
	YTHDC1	regulate mRNA splicing and functions as a mediator in nuclear export	[54–56]
	YTHDC2	improves the translating efficiency of target mRNAs	[57]
	IGF2BP1/2/3	enhance the steadiness, storage and translation efficiency of mRNAs	[49, 58, 59]
	HNRNPA2B1	enhances pri-miRNA processing	[61]
	HNRNPC/G	mediate mRNA abundance and splicing	[2, 62]
	eIF3	promotes mRNA translation	[63, 64]

## m6A in cancer metabolic pathways

Increasing evidence shows that reprogrammed cellular metabolism is a main hallmark of cancer, in addition to the typical characteristics of tumor cell evasion of proliferation inhibitors, escape from immune attack, and capability of proliferation infinity; tumor-induced inflammation, invasion and metastasis; vascular leakage; genomic mutation; resistance to cell death and active proliferative signaling [65, 66]. A recent study reported that nonmutational epigenetic recombination, cellular senescence, phenotypic plasticity and polymorphic microbiomes are also hallmarks of cancer [67]. As one of the most noted characteristics in tumors, aberrant metabolism is, in part, critical for tumorigenesis and cancer progression. Various studies have verified that metabolic reprogramming is also a key factor in modulating resistance to chemotherapy [68, 69]. Tumor cells mainly take advantage of four metabolic pathways, aerobic glycolysis, altered fatty acid metabolism, glutamine-dependent anaplerosis and mitochondrial metabolism, to support biosynthesis and bioenergy metabolism [70, 71]. As the most prevalent RNA modification, m6A exerts widespread effects on cancer metabolism reprogramming by regulating a wide range of metabolic enzymes.

## m6A in glucose metabolism pathways

In the 1950s, Warburg observed that glycolysis is highly activated in the presence of oxygen in cancer cells a process called aerobic glycolysis or the Warburg effect [24]. Depending on this typical glucose metabolism pathway, tumor cells can better adapt to hypoxic conditions to undergo malignant proliferation.

Glucose transporter 1 (GLUT1) is an important transporter in glycolysis, importing glucose into the cytoplasm. In gastric cancer (GC), the long noncoding RNA LINC00958 is highly expressed compared to normal gastric tissues and upregulated level of LINC00958 clinically indicates the poor survival of GC patients. The mechanism is that the m6A methyltransferase KIAA1429 catalyzes LINC00958 which stabilizes GLUT1 and promotes GLUT1 mRNA stability, thereby augmenting the effect of aerobic glycolysis of gastric cancer (GC) [72]. In colorectal cancer (CRC), METTL3 promotes GLUT1 translation to induce glucose uptake and lactate production and activates mammalian target of rapamycin complex 1(mTORC1) signaling, which results in cancer development [73]. High expression of ALKBH5 has been observed in breast cancer tissues from human epidermal growth factor receptor 2 (HER2)-therapy resistant patients. Mechanistic research has revealed that ALKBH5 targets GLUT4, ensuring its mRNA stability and facilitating glycolysis in breast cancer cells [74].

Hexokinase 2 (HK2) is an important enzyme in aerobic glycolysis, controlling the rate of glycolysis pathway activation. The m6A methyltransferase METTL3 links to the 3’-untranslated region of HK2 mRNA and recruits YTHDF1 to ensure HK2 stability, ultimately boosting aerobic glycolysis in cervical cancer (CC) cells, contributing to their proliferation and leading to poor prognosis in cervical cancer [75]. Glutamate also plays a role in pancreatic ductal adenocarcinoma by upregulating METTL3 activity and further promoting HK2 in an m6A dependent manner, ultimately enhancing the glycolysis rate [76]. The writer WTAP, playing functioning as an oncogene in gastric cancer and showing mechanistic action similar to that of METTL3, can bind the 3’-UTR m6A site of HK2, enhance its stability and augment the Warburg effect [77]. In lung cancer, sevoflurane inhibits tumor progression and aerobic glycolysis by indirectly reducing HK2 stability in a manner dependent on m6A [78]. In CRC cells, METTL3 is an oncogene that stabilizes HK2 as well as GLUT1 expression via IGF2BP2- or IGF2BP2/3-related mechanisms and activates the subsequent aerobic glycolysis pathway to boost colorectal cancer progression [79]. Additionally, in CRC cells, KIAA1429 increases HK2 levels to boost— aerobic glycolysis rate [80]. In addition to m6A writers, one of the m6A readers, YTHDF1, is closely related to cancer glucose metabolism. Based on the analysis of lncRNA expression data from gene expression omnibus (GEO) databases, it was discovered that Human Recombinant Protein 5 (HCP5) is significantly upregulated in esophageal squamous cell carcinoma (ESCC) and indicates unfavorable survival of ESCC patients. In mechanism, HCP5 directly interacts with YTHDF1, which subsequently promotes the binding of YTHDF1 to HK2 mRNA, enhances HK2 stability and thereby promotes ESCC progression [81].

Enolase 1 (ENO1), which catalyzes the synthesis of phosphoenolpyruvate (PEP) from 2-phosphoglycerate, plays a key role in promoting aerobic glycolysis. C5aR1-positive neutrophils reinforce aerobic glycolysis to drive breast cancer progression via m6A methylation of ENO1. The cascade involves C5aR1-positive neutrophils secreting IL-1β and TNF-α, thereby activating ERK1/2 signaling, leading to the phosphorylation of the m6A writer WTAP at serine341, which stabilizes it, thereby augmenting the abundance of ENO1 m6A methylation and facilitating glycolysis [82]. In lung adenocarcinoma (LUAD), the antagonism between the m6A writer METTL3 and eraser ALKBH5 is correlated with ENO1-dependent glycolysis. Due to the upregulation of METTL3 and downregulation of ALKBH5, an elevated m6A level on ENO1 suggests a bleak prognosis for LUAD patients because it stimulates glycolysis [83].

Similar to HK2, PKM2 catalyzes one of the rate-determining steps (RDSs) in glycolysis, producing pyruvate derived from phosphoenolpyruvate via substrate-level phosphorylation. The m6A eraser FTO has been demonstrated to show tumorigenic features in hepatocellular carcinoma (HCC). The Cancer Genome Atlas (TCGA) dataset shows that FTO is overexpressed in HCC, which is consistent with clinical data. In addition, the prognosis analysis reveals that high level of FTO indicates the poor survival of HCC patients. Mechanistically, FTO induces the demethylation of PKM2 mRNA to fuel translation, ultimately resulting in HCC [84]. In addition, the m6A writer YTHDF2 enhances PKM2 and subsequently augments aerobic glycolysis in breast cancer [85].

**Fig. 1:**
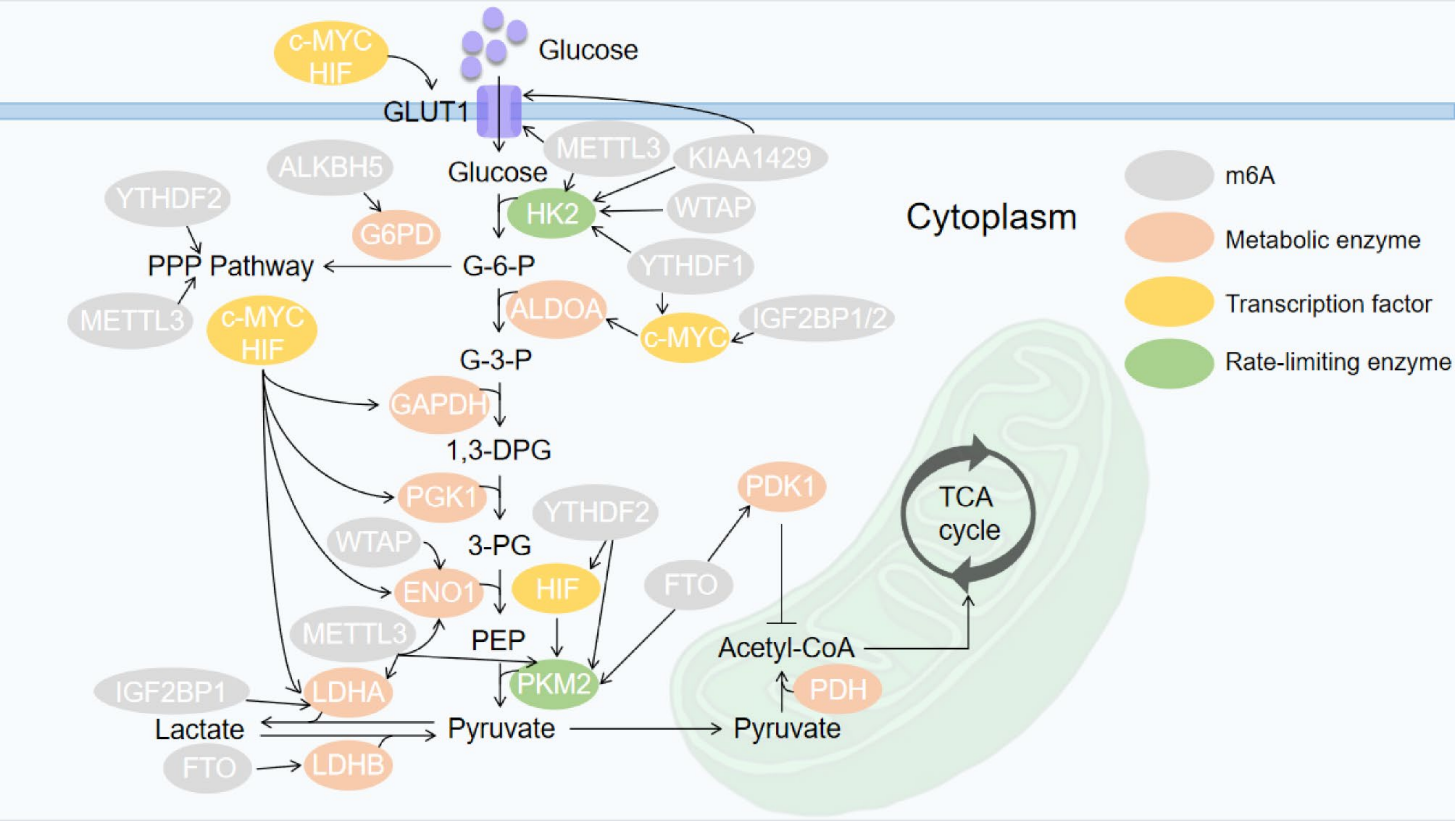
The function of m6A in cancer glucose metabolic pathways

Lactate dehydrogenase A (LDHA) is a critical glycolytic enzyme that accelerates the dehydrogenation reaction of pyruvate conversion to lactic acid, while LDHB can reverse this process because of its higher affinity for lactate [86]. Recently, it was reported that METTL3 can upregulate the level of LDHA to trigger aerobic glycolysis in CRC, especially 5-FU-resistant CRC cells [87]. The landscape analysis based on TCGA shows that IGF2BP1 level is increased in clear cell renal cell carcinoma (ccRCC) and high expression of IGF2BP1 is closely correlated with the lower survival of ccRCC patients. IGF2BP1 participates in clear cell renal cell carcinoma (ccRCC) by recognizing sites on LDHA mRNA modified with m6A marks, increasing LDHA mRNA stability and accelerating aerobic glycolysis[88]. Moreover, R-2-hydroxyglutarate (R-2HG) specifically abrogates the upregulation of LDHB gene expression through m6A eraser FTO demethylation and thereby suppresses glycolysis in leukemia, indicating a function for m6A in cancer metabolic pathways [89] (Fig. 1).

m6A can directly influence glucose transporters or metabolic enzymes to modulate glucose uptake and glycolysis in various cancers. Moreover, other enzymes that regulate glucose metabolism in tumors may be targets of m6A modification. For instance, it is demonstrated that ALKBH5 is downregulated in bladder cancer cells and predicts poor prognosis of bladder cancer individuals. ALKBH5 mediates glycolysis by attenuating the stability of casein kinase 2α (CK2α) in an m6A-dependent manner and thus suppressing the role of CK2α in the glycolytic pathway. The weakened function of CK2α can inhibit the multiplication of bladder cells and thus suppress tumor development [90]. Moreover, a series of studies confirmed that pyruvate dehydrogenase kinase 1 (PDK1) [91], pyruvate dehydrogenase kinase 4 (PDK4) [92], bromodomain PHD finger transcription factor (BPTF) [93], MYC proto-oncogene (MYC) [94–97] and hypoxia-inducible factor (HIF) [98–100] promoted glycolysis in tumor progression in an m6A-dependent manner. The detailed mechanisms are presented in Table 2.

**Table 2:** m6A in cancer metabolic pathways

Metabolism	Related Molecule	Cancer Type	Mechanism	Reference
Glucose metabolism	GLUT1	Gastric cancer	KIAA1429 catalyzes long noncoding RNA Linc00958 which stabilizes GLUT1 in a m6A-dependent manner, thereby augmenting the effect of aerobic glycolysis.	[72]
	GLUT1	Colorectal cancer	METTL3 directly targets the m6A-GLUT1-mTORC1 axis to prompt glucose uptake and lactate production.	[73]
	GLUT4	Breast cancer	ALKBH5 targets at GLUT4 mRNA, promotes its demethylation and stability, and augments glycolysis.	[74]
	HK2	Cervical cancer	METTL3 links to the 3’-Untranslated Region of HK2 mRNA and recruits YTHDF1 to ensure HK2 steadiness, finally facilitating proliferation and aerobic glycolysis.	[75]
	HK2	Pancreatic cancer	Glutamate upregulates METTL3 and further promotes HK2 in m6A dependent manner and ultimately enhances glycolysis.	[76]
	HK2	Gastric cancer	WTAP binds the 3’-UTR m6A site of HK2, enhances its stability and augments the Warburg effect.	[77]
	HK2, GLUT1	Colorectal cancer	METTL3 stabilizes HK2 and GLUT1 expression via IGF2BP2 or IGF2BP2/3 mechanism, and activate the subsequent aerobic glycolysis pathway.	[79]
	HK2	Colorectal cancer	KIAA1429 increases HK2 level so as to boosts aerobic glycolysis.	[80]
	HK2	Esophageal squamous cell carcinoma	YTHDF1 binds to HK2 mRNA to enhance its stability and thereby promotes esophageal squamous cell carcinoma.	[81]
	ENO1	Breast cancer	C5aR1-positive neutrophils secrete TNF-α, activate ERK1/2 signaling, phosphorylate WTAP and ensure its steadiness, thereby augmenting the level of ENO1 m6A methylation to facilitate glycolysis.	[82]
	ENO1	Lung adenocarcinoma	The up-regulation of METTL3 and down-regulation of ALKBH5 lead to the elevated m6A level of ENO1 and stimulate glycolysis.	[83]
	PKM2	Hepatocellular carcinoma	FTO induces the demethylation of PKM2 mRNA to fuel translation.	[84]
	PKM2	Breast cancer	YTHDF2 enhances PKM2 and subsequently augments aerobic glycolysis.	[85]
	LDHA	Colorectal cancer	METTL3 up-regulates the level of LDHA to trigger aerobic glycolysis.	[87]
	LDHA	Clear cell renal cell carcinoma	IGF2BP1 recognizes LDHA m6A sites, increases LDHA mRNA stability and accelerates aerobic glycolysis.	[88]
	LDHB	Leukemia	R-2HG abrogates the upregulation of LDHB gene expression through FTO demethylation and thereby suppresses glycolysis.	[89]
	CK2α	Bladder cancer	ALKBH5 mediates glycolysis by attenuating the stability of CK2α in m6A-dependent manner.	[90]
	PDK1	Glioblastoma	LncRNA just proximal to XIST (JPX) enhances FTO-mediated PDK1 mRNA demethylation to facilitate glycolysis.	[91]
	PDK4	Cervical cancer, Liver cancer	YTHDF1 and IGF2BP3 positively modulates glycolysis by binding with PDK4.	[92]
	BPTF	Renal cell carcinoma	METTL14-mediated m6A modification downregulates the BPTF mRNA stability and induces glycolytic reprogramming.	[93]
	MYC	Cervical cancer	Human papillomavirus (HPV) regulates IGF2BP2 to stabilize the expression of MYC, thus promoting aerobic glycolysis.	[94]
	MYC	Colorectal cancer	Long intergenic noncoding RNA for IGF2BP2 stability (LINRIS) augments the effect of IGF2BP2, and thereby promotes MYC-mediated glycolysis.	[95]
	MYC	Non-small cell lung cancer	YTHDF1 and m6A-modified lncRNA discs large homolog associated protein 1 (DLGAP1) promotes glycolysis by stabilizing c-myc mRNA.	[96]
	MYC	Gastric cancer	IGF2BP1 stabilizes c-myc mRNA and speeds up aerobic glycolysis.	[97]
	HIF-1α	Hepatocellular carcinoma	HBXIP increases m6A abundance of HIF-1α via METTL3, thus driving glycolysis.	[98]
	HIF-2α	Renal cell carcinoma	Methylenetetrahydrofolate Dehydrogenase 2 (MTHFD2) controls the level of m6A in HIF-2α mRNA which promotes the aerobic glycolysis.	[99]
	HIF-1α	Renal cancer	YTHDF2 together with PBRM1 up-regulates HIF-1α to promotes cancer progression.	[100]
	G6PD	Colorectal cancer	YTHDF2 degrades circ-0003215 and further modulates the level of DLG4, which blocks the PPP via the ubiquitination of G6PD.	[101]
	G6PD	Colorectal cancer	METTL3 degradation promotes the stability of LINC01615, upregulates the level of G6PD by enhancing G6PD pre-mRNA splicing and further activates PPP.	[102]
	G6PD	Glioma	ALKBH5 facilitates the mRNA stability of G6PD, enhances its translation and finally stimulates cell proliferation.	[103]
	6PGD	Lung cancer	By recognizing m6A site on 6PGD, YTHDF2 directly binds with 6PGD, promotes its expression and facilitates the progress of lung cancer.	[104]
Lipid metabolism	ACLY, ACC1	Esophageal cancer	HNRNPA2B1 up-regulates ACLY and ACC1 gene expression to fuel fatty acid metabolism.	[113]
	ACLY, SCD1	Hepatocellular carcinoma	METTL3/14 targets at ACLY and SCD1, increases their expression and promotes lipid metabolism.	[114]
	FASN	Hepatocellular carcinoma	FTO ensures the stability of FASN mRNA and prevents mRNA degradation to positively influence lipid metabolism.	[115]
	SREBP	Hepatocellular carcinoma	FTO acts on SREBP1C to affect the downstream effectors FASN, SCD, ACC1, DGAT2, CIDEC and CPT1, promoting lipid synthesis, lipid store and fatty acid oxidation eventually.	[116–120]
	ACSL4	Hepatocellular carcinoma	METTL5 targets at 18 S rRNA, impairs 80 S ribosome, reduces the level of proteins related to fatty acid metabolism. ACSL4 regulates the role of METTL5 in fatty acid metabolism and thus facilitates cancer progression.	[121]
	ACC1	Cervical squamous cell carcinoma	ALKBH5 and IGF2BP1 target at SIRT3, lower its stability, consequently inhibit ACC1 deacetylation and lipid metabolism.	[122]
Amino acid metabolism	SLC1A5	Clear cell renal cell carcinoma	FTO and VHL can indirectly target SLC1A5 in the downstream so as to promote metabolic recombination.	[126]
	GLS	Colon cancer	YTHDF1 targets the 3’ UTR of GLS1 to boost the function of GLS1 and facilitate glutamine metabolism.	[127]
	MYC, GPT2, and SLC1A5	Acute myeloid leukemia	IGF2BP2 targets at MYC, GPT2, and SLC1A5 and enhances glutamine metabolism.	[128]
	BCAT1, BCAT2	Acute myeloid leukemia	METTL16 affect branched-chain amino acid metabolism in AML by upregulating the expression of BCAT1 and BCAT2 via m6A modification.	[129]
Mitochondrial metabolism	PGC-1α	Clear cell renal cell carcinoma	FTO decreases the abundance of m6A in PGC-1α mRNA transcripts, increases its expression and recovers mitochondrial activity.	[130]
	AK4	Breast cancer	METTL3 selectively targets the 5’ UTR of AK4 mRNA, which ultimately inhibits mitochondrial apoptosis.	[132]
	TLR4	multiple myeloma	HNRNPA2B1 enriches at the m6A sites of TLR4, thus enhancing mitochondrial metabolism.	[133]

In addition to aerobic glycolysis, branching glucose metabolic pathways, such as the pentose phosphate pathway (PPP), play important roles in the proliferation of tumor cells. m6A exerts an impact on the PPP to indirectly affect cancer metabolism. Numerous studies have shown that circ-0003215 inhibits tumor progression and metastasis. In CRC cells, YTHDF2 degrades circ-0003215 and further modulates the level of discs large MAGUK scaffold protein 4 (DLG4), which blocks the PPP via the ubiquitination of glucose-6-phosphate dehydrogenase (G6PD) [101]. Under serum starvation conditions, autophagy-induced METTL3 degradation promotes the stability of LINC01615. Thus, the overexpression of LINC01615 upregulates the level of G6PD by enhancing G6PD pre-mRNA splicing and further activates the PPP [102]. ALKBH5 expression is significantly increased in glioma cells and is involved in glioma cell proliferation. In the PPP, ALKBH5 facilitates the mRNA stability of G6PD, enhances its translation and ultimately stimulates cell proliferation [103]. In addition to G6PD, m6A targets 6-phosphogluconate dehydrogenase (6PGD), a cytosolic enzyme in the PPP, to influence cancer glucose metabolism. By recognizing the m6A site on 6PGD, YTHDF2 directly binds with 6PGD, promotes its expression and facilitates the progression of lung cancer [104].

Tricarboxylic acid cycle (TCA cycle), as the downstream pathways of glycolysis, is largely inhibited in primary solid tumor, which is one of the hallmarks of tumor. As shown in the previous studies, m6A modification plays an important role in glucose metabolism. In fact, the interaction between glucose metabolism and m6A is actually mutual. Although the study on the role of m6A in TCA cycle is limited, it is reported that the accumulation of intermediate products in TCA cycle can in turn regulate m6A modification. The lack of succinate dehydrogenase complex (SDH), an essential metabolic enzyme complex in TCA cycle, is quite common in gastrointestinal stromal tumors, which results in the abnormal accumulation of succinate, while succinate can effectively suppress α-ketoglutarate-dependent dioxygenase family enzymes which include m6A modification [105].

## m6A in lipid metabolism pathways

Previous evidence has verified that lipid metabolism is deregulated in malignant tumors [106–108]. Elevated levels of de novo synthesis and altered fatty acid uptake and catabolism are the typical phenotypes acquired during lipid metabolic reprogramming, which satisfies the material needs for the proliferation of tumor cells and is conducive to cancer progression [109–112]. Mechanistically, the m6A modification exerts an effect on relevant metabolic enzymes and regulators to modulate lipid metabolism in cancer.

In lipid metabolism, ATP citrate lyase (ACLY) catalyzes the conversion of cytoplasmic citrate to acetyl-CoA. Subsequently, acetyl-CoA carboxylase-alpha (ACC1) catalyzes the ATP-dependent carboxylation of acetyl-CoA and then produces malonyl-CoA, which is consumed during fatty acid synthesis. Omics research shows that HNRNPA2B1 is substantially increased in esophageal cancer (ESCA) and the prognosis is worse in patients with a high level of HNRNPA2B1. HNRNPA2B1 upregulates ACLY and ACC1 gene expression during fatty acid metabolism to promote esophageal cancer (ESCA) progression, while the knockdown of HNRNPA2B1 suppresses the growth, metastasis and invasion of tumor cells [113]. In addition, the dysregulation of the m6A writers METTL3 and METTL14 is closely connected with HCC development. Mechanistically, METTL3/14 target ACLY and stearoyl-CoA desaturase 1 (SCD1), increase their expression, promote lipid metabolism and ultimately lead to HCC [114].

In HepG2 cells, the first m6A eraser to be identified, FTO, regulates lipogenesis via fatty acid synthase (FASN) in the final step of de novo synthesis in an m6A-dependent manner. By knocking down FTO, the expression of the m6A reader YTHDF2 and the abundance of m6A on FASN mRNA were markedly increased, leading to the instability and ultimate decay of FASN mRNA. As FASN is positively correlated with de novo lipogenesis, the reduced level off FASN eventually results in deficient lipid accumulation [115]. In addition, FTO modulates lipid metabolism in HepG2 cells by acting on the transcription factor sterol regulatory element-binding protein (SREBP) 1 C to affect the downstream effectors FASN, stearoyl-CoA desaturase 1 (SCD1), ACC1, diacylglycerol acyltransferase 2 (DGAT2), cell death-inducing DFFA (DNA fragmentation factor-α)-like effector c (CIDEC) and carnitine palmitoyl transferase 1 (CPT1), eventually promoting lipid synthesis, lipid storage and fatty acid oxidation [116–120].

In HCC, METTL5 has been confirmed to promote tumorigenesis by targeting 18 S rRNA, impairing the 80 S ribosome, and reducing the levels of proteins related to fatty acid metabolism. Moreover, acyl-CoA synthetase long-chain family member (ACSL4) regulates the function of METTL5 in fatty acid metabolism and thus facilitates cancer progression[121].

In cervical squamous cell carcinoma (CESC), ALKBH5 and IGF2BP1 target silent mating type information regulation 2 homolog 3 (SIRT3), reducing its stability, subsequently inhibiting ACC1 deacetylation and lipid metabolism and ultimately repressing CESC[122].

## m6A in amino acid metabolism pathways

An increasing number of studies have documented that cancer cells exhibit a higher demand for amino acids to maintain their vitality and proliferation. Amino acids are, on the one hand, substrates for biosynthesis, and on the other hand, they can be converted into α-ketoglutarate (α-KG) and other metabolites, which together constitute the basic process of amino acid metabolism in tumors. Studies showing that the m6A modification can exert a direct impact on relevant metabolic enzymes or metabolites in amino acid metabolism pathways are relatively rare. However, m6A acts on other significant factors, such as MYC [123], to mediate metabolic reprogramming in cancer.

**Fig. 2:**
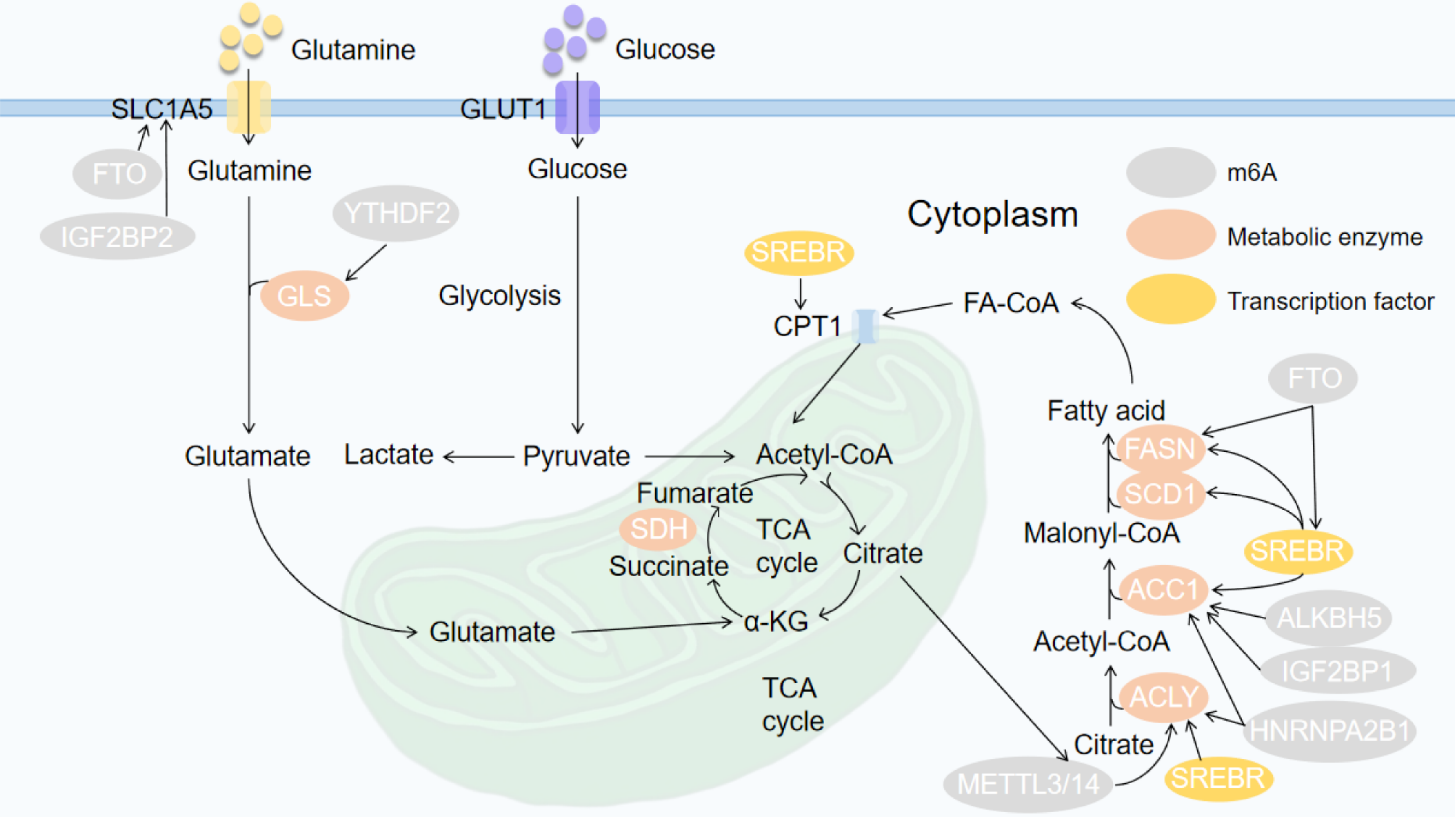
The function of m6A in cancer lipid metabolic and amino acid metabolic pathways

SLC1A5 is an important transporter in amino metabolism pathways that imports glutamine into the cytoplasm [124]. After glutamine is transported, glutaminase (GLS) converts it to glutamate in the first step of glutamine catabolism [125]. The m6A RNA eraser FTO and von Hippel‒Lindau (VHL), a tumor-suppressing factor that is not expressed in ccRCC cells, are synthetic lethality-inducing partners that indirectly target SLC1A5 downstream to promote metabolic recombination [126]. The m6A reader YTHDF1 targets the putative binding motif in the 3’ UTR of GLS1 to boost the function of GLS1, facilitate glutamine metabolism and contribute to the progression of cisplatin-resistant colon cancer [127]. By analyzing TCGA datasets, IGF2BP2 is overexpressed in AML and acts an adverse prognostic factor for AML patients. IGF2BP2 is an adverse prognostic factor for AML patients. IGF2BP2 promotes AML progression by targeting several significant factors in the glutamine metabolism pathways, including MYC, glutamic-pyruvic transaminase 2 (GPT2), and SLC1A5 [128]. Recently, METTL16 was found to affect branched-chain amino acid metabolism in AML by upregulating the expression of branched-chain amino acid transaminase 1 (BCAT1) and BCAT2 mediated via m6A modification [129]. (Fig. 2)

## m6A in mitochondrial metabolism

Glycolysis is one of the most notable and widely studied metabolic processes in all kinds of malignant tumors. Nevertheless, mitochondria are also key elements providing energy for the proliferation of cancer cells in addition to oncogenesis, regulating tumor anabolism, redox and calcium homeostasis, etc. [70]. Therefore, the role of mitochondrial metabolism in cancer cannot be ignored.

Peroxisome proliferator-activated receptor gamma coactivator 1α (PGC-1α) is a transcription coactivator in mitochondrial metabolism. FTO plays an indispensable antitumorigenic role in ccRCC by reducing the abundance of m6A on PGC-1α mRNA, increasing transcript levels and leading to the recovery of mitochondrial activity [130].

Adenylate kinase 4 (AK4) has been demonstrated to indirectly modulate mitochondrial metabolism probably via ADP/ATP translocase, thereby enhancing energy homeostasis [131]. In tamoxifen-resistant breast cancer cells, METTL3 selectively targets the 5’ UTR of AK4 mRNA, which ultimately inhibits mitochondrial apoptosis and enhances tamoxifen resistance [132].

Toll-like receptor 4 (TLR4) signaling plays an important role in multiple myeloma (MM) by maintaining cancer mitochondrial metabolism. Clinical data show that HNRNPA2B1 indicates poor prognosis in MM patients. Mechanistically, HNRNPA2B1 can enhance this process by enriching at the m6A sites of TLR4, thus facilitating cancer progression[133].

## m6A in cancer targeted therapy

Under normal physiological conditions, the m6A modification is a homeostatic process, while m6A dysregulation is involved in cancer cell metabolism, indicating that m6A-targeted therapy may play an important role in inhibiting tumor progression.

## m6A-targeted anti-tumor treatment

Previous studies demonstrated that m6A enzymes were differently expressed in numerous cancers and the abnormal level of m6A in metabolic pathways predicted the poor prognosis of cancer patients. Based on mechanisms of m6A in cancer metabolism, m6A-targeted therapy may provide an insight into the study of targeted anti-tumor treatment. Strategies include: establishing m6A gene-editing system to achieve the knockdown or knockout of oncogenes and the overexpression of tumor suppressor genes, for example clustered regularly interspaced short palindromic repeats associated protein 9 (CRISPR-Cas9) or short hairpin RNA (shRNA) encapsulated by adeno-associated virus (AAV); using nanoparticles to deliver small interfering RNA for targeting m6A enzymes; screening small molecule compounds that specifically regulate m6A; and developing specific and efficient m6A-targeted inhibitors.

In CRC, METTL3 is significantly upregulated and promotes glucose uptake via targeting at metabolic enzymes in glycolysis. It is found that METTL3 knockout by using lentiviral-based CRISPR gene editing system markedly inhibits tumorigenesis in mice, and this may be due to decreased HK2 and GLUT1 level and the reduced hexokinase activity [79]. In vivo experiments confirm that METTL3 knockdown via shRNA has anti-tumor effect in liver cancer. The knockdown of METTL3 significantly attenuates PDK4 mRNA stability, reduces glucose uptake and lactate production and accelerates mitochondrial oxidative respiration [92]. Similarly, METTL3 is significantly upregulated in cervical cancer and promotes glucose uptake via targeting at metabolic enzymes in glycolysis. The knockdown of METTL3 via shRNA induces reduced lactate production and ATP level in vivo and suppresses tumorigenesis [75]. After METTL5 knockout, treatment with ACSL4 knockdown by using siRNA significantly represses fatty acid metabolism of HCC in mice. To be specific, METTL5 knockout and ACSL4 knockdown synergistically reduce the levels of free fatty acids, triglycerides and intracellular lipid droplets and then blocks tumor initiation in HCC tissues [121]. In AML in vivo experiments, the lentiviral vector-based shRNA system was applied to knock down METTL16 in mice, and it was demonstrated that METTL16 knockdown largely inhibited tumorigenesis and development. Consistently, METTL16 knockout also exhibited anti-tumor effect by suppressing the expression of BCAT1 and BCAT2 in amino acid metabolism [129]. KIAA1429 overexpression is associated with the malignancy maintenance of CRC cells. Conversely, KIAA1429 knockdown via shRNA in xenograft model represses tumor growth, which leads to reduced glucose uptake and lack of ATP production [80].

In addition to m6A writers, targeting m6A erasers can also inhibit cancer progression by regulating metabolic pathways. In papillary thyroid cancer (PTC), the overexpression of FTO via lentiviruses containing complete FTO coding sequence suppresses tumor growth in xenograft model. GLUT1, HK2 and LDHA levels are markedly reduced, which ultimately leads to weakened aerobic glycolysis [134]. High expressions of FTO and glutamine transporter SLC1A5 are closely correlated with glutamine metabolism and the poor prognosis of ccRCC patient, while FTO knockdown decreases the survival of ccRCC cells, therefore targeting FTO is a possible anti-tumor therapy. Using shRNA to knockdown FTO, it is found that the growth of ccRCC tumor is significantly decreased in xenograft model. The levels of SLC1A5 and glutamine uptake are reduced upon FTO knockdown [126]. There are various m6A enzymes that participate in cancer metabolism, which forms a complicated regulating network, but it is clear that the abnormal global level of m6A is closely correlated with tumor initiation and progression. In LUAD, high global m6A level is observed due to METTL3 upregulation and ALKBH5 downregulation, and this leads to elevated PEP, pyruvate and ATP levels, while METTL3 knockout and ALKBH5 overexpression exhibit synergistic effect on tumor inhibition in LUAD mouse models [83].

After bone marrow transplantation, IGF2BP2 knockdown via shRNA dramatically decreased the level of immature blast cells in peripheral blood, substantially delayed leukemogenesis and development, and obviously prolonged the lifespan of mice. Moreover, CWI1-2 was identified as a small-molecule IGF2BP2 inhibitor. Treatment with CWI1-2 effectively inhibited AML initiation in vivo and this inhibitor exhibited synergistic effects with other AML chemotherapy, including daunorubicin and homoharringtonine. IGF2BP2 knockdown resulted in the reduced stability of GPT2 and SLC1A5, suppressed amino acid metabolism and decreased levels of metabolites in glycolysis and TCA cycle [128].

Recently, siRNA encapsulated by small extracellular vesicles (sEV) was used to deplete YTHDF1 in GC. It was revealed that YTHDF1 depletion suppressed tumor development and metastasis in vivo. YTHDF1 loss modulated immune regulation in GC by increasing interferon (IFN)-γ receptor 1 expression to promote IFN-γ response and upregulating major histocompatibility complex class I to enhance cytotoxic T lymphocyte reaction [135]. (Table 3)

**Table 3:** m6A-targeted anti-tumor treatment

Cancer Type	m6A	Treatment	Reference
Colorectal cancer	METTL3	METTL3 knockout via lentiviral-based CRISPR gene editing system decreases HK2 and GLUT1 level and the reduces hexokinase activity to inhibit tumorigenesis.	[79]
Liver cancer	METTL3	The knockdown of METTL3 via shRNA attenuates PDK4 mRNA stability, inhibits glycolysis and exhibits anti-tumor effect.	[92]
Cervical cancer	METTL3	The knockdown of METTL3 via shRNA induces reduced lactate production and ATP level and suppresses tumorigenesis.	[75]
Hepatocellular carcinoma	METTL5	METTL5 depletion and siACSL4 reduce the levels of free fatty acids, triglycerides and intracellular lipid droplets and blocks tumor initiation.	[121]
Colorectal cancer	KIAA1429	KIAA1429 knockdown via shRNA represses tumor growth, which leads to reduced glucose uptake and lack of ATP production.	[80]
Acute myeloid leukemia	IGF2BP2	IGF2BP2 knockdown via shRNA and IGF2BP2 inhibition by CWI1-2 delay leukemogenesis and development.	[128]
	METTL16	METTL16 knockdown via shRNA suppresses the expression of BCAT1 and BCAT2 in amino acid metabolism to inhibit tumorigenesis and development.	[129]
Papillary thyroid cancer	FTO	FTO overexpression via lentiviruses containing complete FTO coding sequence reduces GLUT1, HK2 and LDHA levels, attenuates glycolysis and suppresses tumor growth.	[134]
Clear cell renal cell carcinoma	FTO	FTO knockdown via shRNA reduces the levels of SLC1A5 and glutamine uptake to inhibit tumor growth.	[126]
Gastric cancer	YTHDF1	YTHDF1 depletion via siRNA encapsulated by sEV modulates immune responses and suppresses tumor development and metastasis.	[135]

## Development of m6A-targeted inhibitors

Since FTO is involved in the occurrence and poor prognosis of various cancers, such as glioblastoma (GBM) and acute myeloid leukemia (AML), FTO inhibitors are thought to be a promising cancer targeted therapy. In 2011, rhein was first discovered to exert inhibitory effects on FTO and ALKBH5 in vitro. Molecular modeling showed that rhein competitively bound to 3-methylthymine (m3T) and 2-oxoglutarate (2OG), active sites of the FTO enzyme, and Fe^2+^, effectively preventing m6A recognition. Therefore, rhein increased the abundance of m6A modification inside cells[136]. Meclofenamic acid (MA) was later found to be another in vitro FTO inhibitor demonstrated to preferentially act on FTO over ALKBH5[137]. Inspired by this discovery, researchers have reported an increasing number of FTO inhibitors, confirming that FTO is a drug-treatable cancer target. Several fluorescein derivatives, with the base name FL1-11, have been designed and can selectively inhibit FTO demethylation in vitro neither by mimicking 2-OG nor by forming an Fe^2+^ chelate[138]. In 2019, a new derivative of MA named FB23-2 was confirmed to upregulate the abundance of methylation marks on genes essential to leukemia, increasing the level of a tumor suppressor protein, and reducing the abundance of a tumor promoting protein, thereby inhibiting the proliferation of AML cells in vitro and in vivo[139]. Compared to FB23-2, two new small-molecule FTO inhibitors, CS1 and CS2, showed higher efficacy in inhibiting the activity of AML cells. CS1 and CS2 bind to the enzymatic reaction center of FTO, blocking its binding to a target gene and thus inhibiting its methyltransferase activity. In addition, CS1 and CS2 inhibit the immune escape of AML cells by targeting leukocyte immunoglobulin-like receptor subfamily B4 (LILRB4), an immune checkpoint protein [140]. Recently, the oxetane-based compounds were shown to inhibit FTO. Among these chemical compounds, FTO-43 N has been reported to significantly inhibit GBM, AML and GC progression and show the an antiproliferative effect similar to that of 5-fluorouracil in GC cells [141].

As mentioned earlier, m6A is a double-edged sword in tumorigenesis and tumor development. An increase or decrease in m6A levels can promote the progression of different tumors. Hence, in addition to inhibitors of the m6A eraser FTO, small-molecule inhibitors of the m6A writers METTL3/14 have garnered increasing attention in recent years. The first selective small-molecule inhibitors of METTT3 to be identified were adenine derivatives, which exhibited high ligand-binding efficiency, shedding light on the study of selective METTL3/14 inhibitors[142]. Based on adenine library screening, UZH1a was designed as a nanomolar inhibitor of METTL3 and showed cell permeability. Importantly, UZH1a lowered the m6A levels in AML cells, osteosarcoma cells and embryonic kidney cells by binding SAM[143]. UZH2, established via optimization of UZH1a, was later confirmed to be another nanomolar inhibitor of METTL3 in AML cells and prostate cancer cells, showing better metabolic stability than UZH1a [144]. STM2457 is a highly specific and selective inhibitor of METTL3/14 that can directly bind to the SAM-binding site in METTL3 and inhibit the activity of METTL3 methyltransferase and its translation, thereby reducing the m6A level in AML cells. STM2457 effectively restrains the expansion of AML cells in vivo, significantly prolonging the survival of mice and exerting no obvious toxic effect [145]. STM2457 is now in the first stage of a clinical trial. Obviously, most of the METTL3/14 inhibitors to date are competitive inhibitors.

Recently, eltrombopag was demonstrated to be a noncompetitive inhibitor that may bind to the allosteric site of METTL3/14, inhibit METTL3/14 complex activity and thus reduce m6A levels in AML cells[146]. Moreover, a previous study verified that eltrombopag prolonged the lifespan of mouse models of AML via antiproliferative and differentiation-inducing effects in vivo[147]. Hence, further optimization of eltrombopag may facilitate new drug development for AML. 4-[2-[5-Chloro-1-(diphenylmethyl)-2-methyl-1 H-indol-3-yl]-ethoxy] benzoic acid (CDIBA), an allosteric inhibitor of METTL3/14, was optimized by combining the best substituents in different regions, and it showed a higher METTL3/14 inhibitory effect than other inhibitors [148]. Although the development of small-molecular drugs targeting METTL3 and METTL14 has slower than that of FTO inhibitors, the study of METTL3/14 inhibitors shows great promise.

## m6A and therapeutic resistance

In fact, m6A enzymes are not only closely correlated with cancer initiation and progression, but also responsible for the therapeutic resistance and altered metabolic pathways in various cancers [149], which may expand the application of m6A-targeted inhibitors. Combination of m6A-targeted inhibitors and chemotherapy may contribute to anti-tumor treatment.

In primary leukemia, FTO increases the stability of a series of proliferation and survival mRNAs, for example B-cell lymphoma-2 (BCL-2) and tyrosine-protein kinase Mer (MERTK), and subsequently promotes protein synthesis. Leukemia cells with the overexpression of FTO demonstrate higher tyrosine kinase inhibitor (TKI) tolerance, while cells exposed to the FTO inhibitor rhein reverses TKI resistance [150]. Interestingly, leukemia cells with the upregulation of FTO are more sensitive to R-2HG by FTO-enhanced stability of MYC transcripts [151]. In CRC, FTO promotes the resistance to 5-fluorouracil (5-FU) and cisplatin. Mechanistically, FTO regulates the expression of G6PD, modulates NADPH and (reactive oxygen species) ROS levels and consequently affects DNA damage. Meanwhile, FTO also regulates the mRNA stability of poly ADP-ribose polymerase 1 (PARP1) to mediate DNA damage repair, which ultimately leads to the reduced chemotherapy sensitivity [152]. In GBM, JPX modulates the expression of PDK1 by interacting with FTO, which prevents PDK1 mRNA from degradation and results in enhanced aerobic glycolysis and the resistance to temozolomide [91]. In addition to chemotherapy resistance, FTO also participates in immunotherapy resistance by regulating glycolysis-associated genes. FTO enhances the expression of c-Jun, JunB, and CCAAT/enhancer binding protein β (C/EBPβ), and then rewires glycolysis metabolism, which impairs the function of T cells and leads to poor immunotherapy response in melanoma. Therefore, the inhibition of FTO is a potential strategy for improving immunotherapy effects [153].

In addition to FTO, other m6A enzymes are also involved in cancer drug resistance via regulating metabolism. METTL3-mediated m6A methylation induces the splicing of estrogen receptor related receptor γ (ERRγ) precursor mRNA, and then ERRγ binds to ATP binding cassette subfamily B member 1 (ABCB1), reinforces its transcription and lowers the sensitivity of cancer cells to numerous anticancer agents. Moreover, ERRγ interacts with the rate-limiting enzyme CPT1B to promote fatty acid metabolism and leads to chemoresistance [120]. In bone marrow mesenchymal stem cells of AML, however, the expression METTL3 is reduced, which enhances adipogenesis and mediates the resistance to penicillin and streptomycin via upregulating AKT protein level [154].

It was revealed that an increased level of ALKBH5 was closely correlated with the glycolysis of breast cancer and the resistance to trastuzumab and lapatinib via the demethylation of GLUT4 and suppression of GLUT4 re-sensitized the resistant cells, indicating that the inhibition of ALKBH5/GLUT4 axis may contribute to breast cancer targeted therapy [74].

YTHDF1 interacts with the binding motif of GLS1 and increases glutamine uptake to enhance glutamine metabolism in cisplatin-resistant CRC cells. The inhibition of GLS1 leads to an increased sensitivity to cisplatin-induced cell death and YTHDF1 is also a potential target in overcoming resistant colon cancers [127]. In gastric cancer, YTHDF2 reduces the stability of cystathionine β-synthase (CBS) mRNA, subsequently decreases the methylation of ACSL4, an important enzyme in lipid metabolism, and results in ACSL4 degradation and chemoresistance. Patients with low CBS levels have a worse prognosis and are less sensitive to chemotherapy [155]. As mentioned above, m6A enzymes participate in chemoresistance via altered metabolic pathways in many different cancers. Hence, m6A inhibitors have great development perspective.

## Conclusion

In the past few years, numerous studies have demonstrated that m6A fuels cancer progression by regulating tumor metabolism. Owing to the development of methods for detecting the m6A modification, numerous studies have revealed a possible role for m6A in aerobic glycolysis, the deregulated metabolism of fatty acids and amino acids and aberrant mitochondrial metabolism. This review analyzes several confirmed functions of the m6A modification in cancer metabolic pathways as well as the underlying mechanisms. In fact, the mutual effects of m6A and cancer metabolic pathways are very complicated. Signaling pathways, transcription factors and noncoding RNAs can exert large impacts on metabolic enzymes in a wide range of cancers, forming a complex network involved in metabolic reprogramming. m6A exerts different influences on cancer metabolism by targeting upstream molecules or downstream metabolic enzymes and transporters, thereby extensively modulating metabolic recombination. Conversely, cancer metabolic reprogramming regulates the abnormal m6A modification. Take glucose metabolism for example, numerous studies confirm that m6A participate in cancer glycolysis, while the accumulation of succinate, an intermediate product in glucose metabolism, can in turn regulate m6A modification. Studies on metabolic enzymes in amino acid metabolic pathways and mitochondrial metabolism are relatively rare, and in-depth investigation is needed to further explain and elucidate the systematic molecular mechanisms involved. There is still a long way to go in the study on the mutual effect of cancer metabolism on m6A modification.

To date, an increasing number of m6A inhibitors have been designed and a series of experiments in vivo and in vitro have confirmed that they have a good application prospect. However, relevant clinical evidence is quite insufficient and clinical trials are urgently needed.

Since dysregulated m6A is closely correlated with tumor development, m6A may also function as specific biomarkers involved in early diagnosis. For instance, multi-omics data suggests that METTL7A is differently expressed in ccRCC, renal mesothelioma and sarcoma, and shows high accuracy in predicting tumorigenesis. Therefore, METTL7A is a potential diagnostic biomarker in certain cancer types [156]. Although accumulating evidence shows that abnormal m6A level may play an important role in the development and progression of cancer, most of m6A-related proteins are located in the nucleus, which cannot be detected in body fluids unless invasive puncture examination is performed. Serological marker, including secretory proteins, exosomes, cytoplasmic tRNAs (ctRNAs), cell-free RNAs (cfRNAs) and some non-coding RNAs, is a better choice for early diagnosis. Actin filament-associated protein 1-antisense RNA1 (AFAP1-AS1) is a lncRNA that encodes AFAP1-AS1 translated mitochondrial-localized peptide (ATMLP) under the control of m6A. The serum level of ATMLP is upregulated in NSCLC, which indicates that ATMLP is a diagnostic marker for lung cancer [157]. In HCC, m6A-modulated circRNAs also function as biomarkers which can be detected in serum, for example circular cleavage and polyadenylation specific factor 6 (circCPSF6), circular mitogen-activated protein kinase kinase kinase 4 (circMAP3K4) and circular fucosyltransferase 8 (circFUT8) [158–160].

In conclusion, m6A can regulate cancer metabolic reprogramming in distinct ways, providing a more comprehensive knowledge of epigenetics and cancer metabolism to better carry out clinical experiments. These studies may be particularly helpful in early diagnosis, disease control and therapeutic approaches to cancer.

## Data Availability

Data sharing not applicable to this article as no data-sets were generated or analyzed during the current study.
